# Physical Interactions and Expression Quantitative Traits Loci Identify Regulatory Connections for Obesity and Type 2 Diabetes Associated SNPs

**DOI:** 10.3389/fgene.2017.00150

**Published:** 2017-10-13

**Authors:** Tayaza Fadason, Cameron Ekblad, John R. Ingram, William S. Schierding, Justin M. O'Sullivan

**Affiliations:** ^1^Liggins Institute, University of Auckland, Auckland, New Zealand; ^2^Plant & Food Research, Auckland, New Zealand

**Keywords:** Obesity and type-2 diabetes co-morbidity, spatial gene regulation, eQTLs, GWAS risk variants, Hi-C

## Abstract

The mechanisms that underlie the association between obesity and type 2 diabetes are not fully understood. Here, we investigated the role of the 3D genome organization in the pathogeneses of obesity and type-2 diabetes. We interpreted the combined and differential impacts of 196 diabetes and 390 obesity associated single nucleotide polymorphisms (SNPs) by integrating data on the genes with which they physically interact (as captured by Hi-C) and the functional [i.e., expression quantitative trait loci (eQTL)] outcomes associated with these interactions. We identified 861 spatially regulated genes (e.g., *AP3S2, ELP5, SVIP, IRS1, FADS2, WFS1, RBM6, HORMAD1, PYROXD2*), which are enriched in tissues (e.g., adipose, skeletal muscle, pancreas) and biological processes and canonical pathways (e.g., lipid metabolism, leptin, and glucose-insulin signaling pathways) that are important for the pathogenesis of type 2 diabetes and obesity. Our discovery-based approach also identifies enrichment for eQTL SNP-gene interactions in tissues that are not classically associated with diabetes or obesity. We propose that the combinatorial action of active obesity and diabetes spatial eQTL SNPs on their gene pairs within different tissues reduces the ability of these tissues to contribute to the maintenance of a healthy energy metabolism.

## Introduction

Genome-wide association studies (GWAS) have been instrumental in identifying numerous genetic risk loci for type 2 diabetes and obesity (reviewed in Pigeyre et al., 2016). Despite this, the identified risk loci for obesity and type 2 diabetes only explain 3 and 10% of the heritability of these disorders, respectively (Sanghera and Blackett, 2012; Pigeyre et al., 2016). Moreover, as most of these variants fall outside of coding regions, they do not have clear biological functions that link them to either obesity or diabetes (Speliotes et al., 2010). Alongside other studies into the pathogenesis of polygenic disorders, this has led to the hypothesis that some of the information within the genome that is responsible for the heritability of diabetes and obesity is not encoded in the linear sequence but instead lies within the spatial organization of the chromatin (Franzen et al., 2016; Schierding et al., 2016). This hypothesis is increasingly supported by empirical evidence that genetic variants fall within regulatory regions (e.g., enhancers, insulators, etc.) that impact on distal, but spatially associated, loci rather than on the genes that are closest to them in the linear DNA sequence (Franzen et al., 2016; Jo et al., 2016; Schierding et al., 2016). Pullinger et al. (2014) for example, have reported an association between a type 2 diabetes variant in *HMGA1* on chromosome 6 and the transcription of the *INSR* gene on chromosome 19.

Proximity-ligation methodologies coupled to high throughput sequencing have enabled a step change in the deconvolution of the spatial organization of genomes. These methods (e.g., genome conformation capture and Hi-C) capture regions of the genome that are physically associated and able to be covalently connected by a cross-linking agent (Grand et al., 2011). Collectively, studies using proximity-ligation methods have begun to untangle how the organization of the genome into non-membrane bound compartments is related to the realization of the information encoded in the DNA sequence itself. This increase in our understanding extends to complex looping patterns that contribute to gene regulation (e.g., reviewed in Pombo and Dillon, 2015).

Spatial chromosomal organization is probabilistic (Bolzer et al., 2005), tissue or cell-type-specific (Parada et al., 2004), developmental stage specific (Krijger et al., 2016; Doynova et al., 2017) and can change to adapt to an evolutionary selective pressure e.g., the inverted genomic structure of rod cells in nocturnal mammals (Solovei et al., 2009). Despite the presence of cell type dependent features, there is remarkable retention of some aspects of chromosomal organization within metazoan nuclei. For example, topologically associating domains (TADs) are remarkably stable across cell types and species (Dixon et al., 2012) and preferential intra-TAD and inter-TAD contacts have been identified (Fraser et al., 2015). Thus, nuclear structure contains both cell type dependent and independent features.

The potential for integrating information on spatial organization and functional data to improve our understanding of the genetic basis of complex phenotypes has been illustrated in several recent studies (e.g., autoimmune Farh et al., 2015, cardiometabolic Franzen et al., 2016, and schizophrenia Won et al., 2016). It is increasingly clear that genetic variants identified by GWAS: (a) can have greater regulatory effects on distant but spatially proximal genes than on the genes closest to them; and (b) can act on more than one gene in a tissue- and developmental-stage specific manner. However, these studies concentrate on cis-regulatory connections and ignore the trans-connections, which were shown to contribute to heritability in human growth (Schierding et al., 2016).

Current approaches to the mapping of genes affected by single nucleotide polymorphisms (SNPs) identified in GWAS typically use the nearest gene model. However, clinical risk for polygenic disorders is the sum result of the gene-environment interactions. These interactions occur within the context of a regulatory network that is “tuned” by the combined action of regulatory sites that spatially cluster. These sites are subject to genetic variation, which may alter these spatial clusters and thereby disrupt the functioning of target genes. As such, it is imperative that previously identified intergenic GWAS variants are tested for spatial interactions. Crucially, this approach has the potential to elucidate the regulatory network that describes the disease-associated SNPs which enhance or reduce the expected co-occurrence and severity of both obesity and type 2 diabetes.

Here we integrate information on spatial organization and functional (i.e., expression) data to identify the overlap between regulatory pathways that contribute to type 2 diabetes, obesity, and comorbid obesity plus type 2 diabetes phenotypes. We demonstrate that loci marked by diabetes—and obesity-related SNPs are involved in regulatory interactions in a tissue—and disease-specific manner.

## Research design and methods

### Identification of regulatory SNP-gene interactions

**Figure 1:**
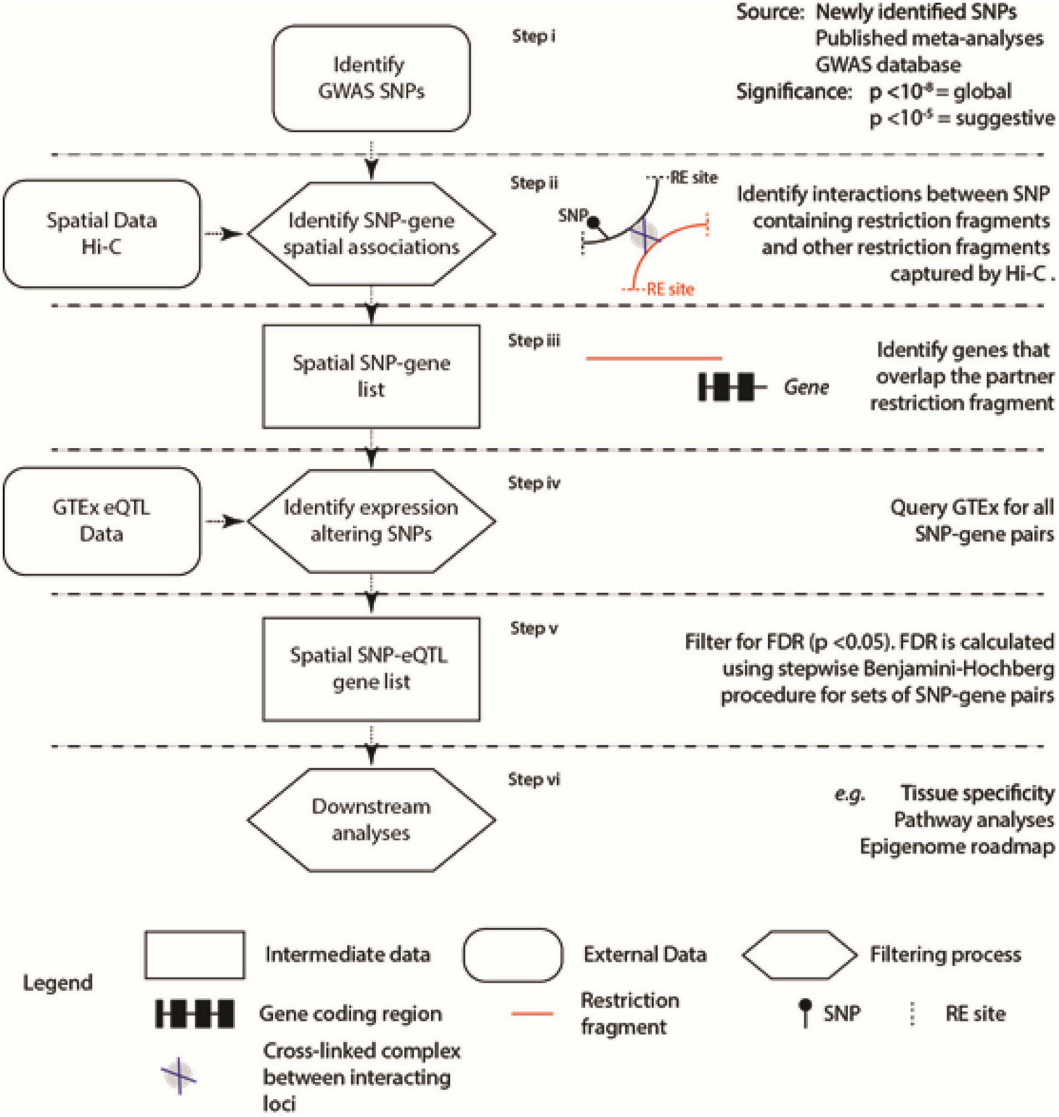
Regulatory SNP-gene interactions that correlate with spatial connections were identified from existing spatial (e.g., Hi-C Rao et al., 2014) and eQTL data (i.e., GTEx Ardlie et al., 2015). Spatial co-localization of the SNP and gene encoding loci is identified from the Hi-C data (Rao et al., 2014) and requires capture of the interaction by proximity-ligation (Step ii). Only genes, determined by the hg19/GRCh37 human genome reference, that overlap the interacting partner locus are included in the analysis. The six stages of the analysis are separated by horizontal dashed lines.

Our aim was to identify SNPs where the genetic variant correlates with the expression level of the spatially associated partner gene [i.e., the SNP is an expression quantitative trait locus (eQTL); Figure 1]. To do this, we developed the “Contextualize Developmental SNPs using 3D Information” (CoDeS3D) algorithm (Figure 1) for the integrated analysis of GWAS SNPs and their phenotypes (GitHub, https://github.com/alcamerone/codes3d).

Creation of a single comprehensive database to interrogate the physical and regulatory interactions between loci, located in cis (<1 Mb apart) and trans (>1Mb apart or on different chromosomes), is prohibited by the complexity and continuing evolution of the relevant datasets (Supplementary Table 1). To overcome this, CoDeS3D is a series of modular Python scripts that uses information on the 3-dimensional organization of the genome (i.e., Hi-C data Rao et al., 2014) to identify spatial connections between regulatory regions, which are marked by SNPs, and the gene(s) that they regulate (Figure 1).

The 3-dimensional structure of genomes can be captured by proximity ligation methodologies (Grand et al., 2011) of which Hi-C is one. High resolution Hi-C data (Rao et al., 2014) was used to identify loci that were captured interacting with restriction fragments containing the SNPs of interest (Figure 1 Step ii). These interactions were identified on a presence/absence basis at the restriction fragment level. This approach identifies interacting loci (as defined by the flanking MboI restriction sites Rao et al., 2014) that are spatially co-localized (Figure 1 Step iii). In some instances this spatial co-localization equates to linear co-localization within the genome sequence. The spatial clustering, however, is not limited to loci that map to adjacent regions within the linear sequence, as trans-spatial interactions are readily identified. Finally, genes (as determined by the hg19/GRCh37 human genome reference) had to overlap the partner locus of interest in order to be included in the list of SNP-gene pairs that were identified (Figure 1 Step iii).

These SNP-gene pairs were then tested against the Genotype-Tissue Expression (GTEx) database (Version 4.1, 09/30/16) to identify those where the identity of the base at the SNP position correlated with a change in the mRNA level of the partner gene (i.e., the SNP was an eQTL). The identification of spatial SNP-gene connections is a tool to filter the test set of eQTL regulatory interactions between the physically connected SNP-gene pairs. This approach reduces the number of tests that need to be performed, compared to a systematic approach to identify both cis- and trans-acting eQTLs. The false-discovery rate (FDR, or *q*-value) is computed for each eQTL SNP-gene-tissue combination, using the *p*-value list, the number of tests performed, and a stepwise Benjamini-Hochberg correction procedure (Benjamini and Hochberg, 1995). Trans-acting eQTL SNPs are selected as significant if the *q*-value is <0.05. Cis-acting eQTL SNPs are selected as being significant according to the calculated threshold for each gene. While our FDR thresholds are less stringent than GTEx (cis *p*-value < 2.5 × 10^−7^; trans *p*-value < 5.0 × 10^−13^), the application of a filtering step to remove large numbers of false positives justifies this, as previous work has identified such thresholds as identifying biologically significant associations(Schierding et al., 2016) and SNPs with p values ≤ 0.05 have clear effects on height (Boyle et al., 2017).

### Ingenuity® pathway analysis

Lists of the genes regulated by loci marked by the eQTL SNPs were analyzed in Ingenuity® Pathway Analysis (IPA®; version 28820210, 2016-09-25) to identify enriched pathways and biological functions.

## Results

### Diabetes and obesity associated SNPs form part of a regulatory network

We reasoned that GWAS SNPs that are associated with type 2 diabetes and obesity mark regulatory loci that modulate spatially proximal genes and function to control energy balance. Using the CoDeS3D pipeline (Figure 1), we were able to identify 45,517 and 27,778 unique pairs of spatial SNP-gene interactions for 1326 and 483 obesity and diabetes SNPs, respectively (Table 1).

**Table 1:** Summary of the regulatory network for the obesity and diabetes SNPs analyzed using CoDeS3D.

	Obesity SNPs^*^		Diabetes SNPs^*^	
	***p* < 5.0E-8**	**5.0E-8 ≤ p ≤ 9.0E-6**	***p* < 5.0E-8**	**5.00E-8 ≤ *p* ≤ 9.0 E-6**
N°. SNPs	186	1,140	183	300
N°. spatial SNP-gene pairs^#^	6,441	39,076	11,344	16,434
N°. eQTL SNPs^†^	76	314	90	106
N°. eGenes^||^	125	444	141	151
N°. eQTL SNP-eGene pairs^§^	148	478	175	177
N°. eQTL SNP-eGene interactions^‡^	690	1,836	605	513
N°. trans eQTL SNP-eGene interactions^¶^	23	84	26	28

* SNPs were identified in GWAS catalog [version, v1·0; download dates (obesity, 2016-07-13; diabetes, 2016-08-26)].

# Spatial SNP-gene pairs were those whose Hi-C restriction fragments overlapped (Figure 1 Step iii).

† eQTL SNPs were defined as having significant (FDR ≤ 0·05) interaction(s) with at least one gene.

|| eGenes were those whose expression was shown to be affected by an eQTL SNP.

§ Non-redundant significant (FDR ≤ 0·05) eQTL SNP-eGene pairs (Figure 1 Step v).

‡ The total number of eQTL SNP-eGene interactions with FDR ≤ 0·05 in at least one GTEx tissue.

¶ *Trans eQTL interactions were defined as occurring between loci > 1Mb apart, or on different chromosomes, with a FDR ≤ 0·05*.

The identification of SNP-gene interactions reduces the number of tests required to detect eQTL SNPs, the sample sizes, and *p*-values required for significance. Therefore, we implemented a stepwise Benjamini-Hochberg correction to select significant eQTLs with a *q* < 0.05. This approach resulted in the identification of 462 cis-acting and 107 trans-acting eQTL SNP-gene pairs for the obesity SNPs, and 238 cis-acting and 54 trans-acting eQTL SNP-gene pairs from the diabetes SNPs (Table 1; Supplementary Figure 1). Obesity eQTL SNP-gene pairs were identified with high significance (*p* < 7.0 × 10^−5^ and *p* < 7.5 × 10^−5^ for the cis- and trans-acting eQTL-SNPs, respectively; Supplementary Spreadsheet 1 doi: 10.17608/k6.auckland.5285038). Similarly, highly significant diabetes cis- and trans-acting eQTL-SNPs were identified (*p* < 6.0 × 10^−5^ and *p* < 6.0 × 10^−5^, respectively; Supplementary Spreadsheet 2 doi: 10.17608/k6.auckland.5285041). Comparisons with the SNP associations in the GWAS catalog indicated that >60% of the eQTL SNP associations have not been previously mapped (Supplementary Figure 2), consistent with previous observations of the accuracy of the “nearest gene” mapping approach for GWAS SNPs (Ardlie et al., 2015).

We used a Monte Carlo method to test for an enrichment for eQTL connections within the diabetes and obesity associated SNPs. eQTLs were identified in 1,000 sets of 483 SNPs that were randomly chosen (with replacement) from a set of 1264: (a) SNPs randomly selected from the Single Nucleotide Polymorphism database (dbSNP build 147, 14/04/2016); and (b) GWAS catalog SNPs that were associated with non-diabetes related traits (Supplementary Table 2). The dbSNP tests resulted in the identification of between 0 and 14 eQTL SNP regions acting on between 0 and 22 genes per set of 483 randomly selected SNPs (Table 2). Notably, the diabetes and non-diabetes associated SNPs identified significantly (*t*-test *p*-value < 0.00001) more connections than the dbSNP tests – consistent with a functional role for the regions labeled with these SNPs in phenotype development. Therefore, we conclude that the number of eQTL SNP-gene pairs we observed is significant (*p* < 0.00001) and unlikely to be due to false positives created from random spatial eQTL connections.

**Table 2:** A Monte Carlo method was used to analyse the eQTL relationships for 1000 sets of 483 SNPs randomly selected from: (a) dbSNP; and (b) non-diabetes associated SNPs.

	**dbSNP**			**Non-diabetes SNPs**		
	**Range**	**Mean**	**StDev**	**Range**	**Mean**	**StDev**
N°. spatial SNP-gene pairs^#^	842–2,297	1,524.38	224.44	24,559–27,550	25911.5	452.71
N°. eQTL SNPs^†^	0–14	5.137	2.77	159–227	191.60	11.63
N°. eGenes^||^	0–22	7.266	4.51	233–380	306.85	24.63
N°. eQTL SNP-eGene pairs^§^	0–22	7.266	4.51	264–436	344.63	29.3
N°. eQTL SNP-eGene interactions^‡^	0–73	27.943	19.73	987–2,001	1,431.76	176.23
N°. trans eQTL SNP-eGene interactions^¶^	0–8	1.46	1.51	33–93	59.84	10.13

*Python's random library was used to randomly select SNPs from dbSNP build 147 (14/04/2016) and non-diabetes associated SNPs from the GWAS Catalog (Supplementary Table 2). The cis- and trans-eQTL regulatory interactions within each set were identified using CoDeS3D*.

# Spatial SNP-gene pairs were those whose Hi-C restriction fragments overlapped (Figure 1 Step (iii).

† eQTL SNPs were defined as having significant (FDR ≤ 0·05) interaction(s) with at least one gene.

|| eGenes were those whose expresssion was shown to be affected by an eQTL SNP.

§ Non-redundant significant (FDR ≤ 0·05) eQTL SNP-eGene pairs (Figure 1 Step v).

‡ The total number of eQTL SNP-eGene interactions with FDR ≤ 0·05 in at least one GTEx tissue.

¶ *Trans eQTL interactions were defined as occurring between loci > 1Mb apart, or on different chromosomes, with a FDR ≤ 0·05*.

Due to the restrictions on Hi-C data resolution, SNPs that were in strong linkage and located on the same restriction enzyme fragment were not separable using CoDeS3D. For example, *IRS1* expression is associated with rs1515110, rs2943640 (*r*^2^ = 0.85), rs2943641 (*r*^2^ = 0.86), rs925735 (*r*^2^ = 0.93), and rs2176040 (*r*^2^ = 0.87; Table 3). All five of these eQTL SNPs mark an *IRS1* regulatory element that is located on a single MboI restriction fragment. Similar effects were observed for the genetic variants that regulate the *FADS1* [i.e., rs174541 and rs174550 (*r*^2^ = 0.89)], *JAZF1* [i.e., rs849134, rs849135 (*r*^2^ = 0.95), rs864745 [*r*^2^ = 0.97]) and *NPC1* [i.e., rs1805081 and rs1808579 (*r*^2^ = 0.7) Table 3]. These examples highlight the inappropriateness of annotating one SNP as being causal, with respect to the eQTL, in the absence of additional information that separates the effects of the combinations of linked variants that are within the restriction fragment.

**Table 3:** Effects of spatial eQTL SNPs on genes involved in lipid metabolism (IPA knowledgebase) that are expressed (RPKM > 1.0) in subcutaneous and visceral adipose, skeletal muscles, and pancreas.

**eQTL SNP (rs^#^)**	**OR/Beta**	**OR/Beta direction**	**Disease/Trait**	**Trait description^¥^**	**GWAS-mapped gene**	**CoDeS3D-mapped gene**	**Effect size^#^**			
							**Adipose subcut·**	**Adipose visceral**	**Skeletal muscle**	**Pancreas**
SNPS: TYPE 2 DIABETES; DISEASE RISK: POSITIVE^*^										
507506	0.022	Decrease	Adiponectin levels		*DLG4*	*ACADVL*	−0.20	–	–	–
507506	0.022	Decrease	Adiponectin levels		*DLG4*	*CLDN7*	−0.36	−0.44	−0.56^§^	–
174541	0.28	Increase	Metabolite levels	adrenate	*FEN1-FADS1*	*FADS1*	–	–	−0.21	−0.70
174550	ND		Fasting glucose-related		*FADS1*	*FADS1*	–	–	−0.20	−0.73
7945071	3.012	Increase	Cognitive function	RAVLT	*LOC105369486*	*FDX1*	–	0.23	–	–
2290402	ND		Type 2 diabetes	AA	*TMEM175*	*IDUA*	–	−0.41	−0.29	–
1515110	0.022	Decrease	Adiponectin levels		*LOC646736-LOC105373913*	*IRS1*	−0.26	–	–	–
2943640	1.09		Type 2 diabetes		*LOC646736-LOC105373913*	*IRS1*	−0.31	–	–	–
2943641	1.19		Type 2 diabetes and other traits		*LOC646736-LOC105373913*	*IRS1*	−0.29	–	–	–
925735	0.02	Decrease	Adiponectin levels		*LOC105373913-LOC105373915*	*IRS1*	−0.27	–	–	–
10510110	1.05		Type 2 diabetes		*PLEKHA1-LOC105378525*	*PLEKHA1*	−0.20	–	−0.14	–
7493	1.06		Yu-Zhi constitution type in type 2 diabetes	Genotype model	*PON2*	*PON2*	–	–	−0.28	−0.37
SNPS: TYPE 2 DIABETES; DISEASE RISK: NEGATIVE^*^										
849134	1.13		Type 2 diabetes		*JAZF1*	*JAZF1*	0.20	0.25	0.27	0.52
849135	1.12		Type 2 diabetes		*JAZF1*	*JAZF1*	0.20	0.25	0.27	0.52
864745	1.1		Type 2 diabetes		*JAZF1*	*JAZF1*	0.20	0.24	0.27	0.51
2290402	ND		Type 2 diabetes	AA	*TMEM175*	*DGKQ*	−0.30	–	–	–
507506	0.022	Decrease	Adiponectin levels		*DLG4*	*CTDNEP1*	0.21	–	–	–
SNPS: OBESITY; DISEASE RISK: POSITIVE^*^										
2230061	0.06	Decrease	Fat body mass	Adjusted by Lean body mass	*CTSS*	*ARNT*	–	0.19	–	–
2230061	0.06	Decrease	Fat body mass	Adjusted by Lean body mass	*CTSS*	*CTSS*	0.17	–	0.29^§^	0.33
8050907	0.03	Increase	Obesity-related traits	Total antioxidants	*PKD2L1*	*HMOX2*	–	–	0.66	–
10540	0.028	Increase	Body mass index	EA, men	*RNH1*	*HRAS*	−0.31	–	−0.24	–
2176040	0.024	Increase	Body mass index	EA, men	*LOC646736-LOC105373913*	*IRS1*	−0.29	–	–	–
4144743	0.023	Increase	Body mass index	EA	*MYL4-ITGB3*	*ITGB3*	−0.33	–	−0.31^§^	–
1805081	1.41		Obesity	Adults	*NPC1*	*NPC1*	0.54	0.45	0.14	0.40
1808579	0.022	Increase	Body mass index	EA, women	*C18orf8, NPC1*	*NPC1*	0.45	0.37	0.13	0.38
4888671	0.03	Increase	Obesity-related traits	Folate	*NUDT7-VAT1L*	*NUDT7*	0.44	0.46	–	0.58
11247915	0.03	Increase	Obesity-related traits	Calorimeter activity	*ALLC, LOC105373393*	*PIGV*	–	–	−0.2	–
10540	0.028	Increase	Body mass index	EA, men	*RNH1*	*PTDSS2*	−0.36	−0.43	−0.36	–
2650492	0.021	Increase	Body mass index	EA	*SBK1*	*SULT1A1*	–	–	−0.29	−0.51
SNPS: OBESITY; DISEASE RISK: NEGATIVE^*^										
17001654	0.03	Increase	Body mass index		*SCARB2*	*NAAA*	0.35	–	–	–
7503807	1.04		Obesity	Overweight	*RPTOR*	*RPTOR*	–	–	0.16	–

# The effect size of the eQTL as defined by GTEx. The slope of the linear regression computed as the effect of the alternative allele (ALT) relative to the reference allele (REF) in the human genome reference GRCh37/hg19 (i.e., the eQTL effect allele is the ALT allele).

§ Gene has expression of RPKM <1 0. RPKM (Reads Per Kilobase of transcript per Million mapped reads) is a measure of the abundance of transcripts in RNA-Seq.

* positive variant, increases risk for disease; negative variant, reduces risk for disease; ambiguous variant, risk is unclear.

¥ *RAVLT, Rey Auditory-Verbal Learning Task; AA, African-Americans; EA East Asians*.

### Co-regulation occurs for localized genes

Obesity and diabetes spatial eQTL SNPs mark loci that co-regulate the genes they are in physical proximity with. The obesity SNP rs2710323, together with two diabetes SNPs, rs2590838 (*r*^2^, 0.78) and rs1108842 (*r*^2^, 0.8), are located in loci that regulate genes (*TMEM110, MUSTN1, ITIH4, NEK4, GNL3, PBRM1*, and *NT5DC2*) within a 300 kb genomic region on chromosome 3 (Supplementary Figure 3). SNPs rs2710323, rs2590838, and rs1108842 are located in a region that upregulates *TMEM110* and down-regulates *NEK4*. Although these three SNPs are in high LD and the genes are close together, the effect of the SNPs is gene-specific. For example, the region marked by rs2590838 is associated with down-regulation of the *NT5DC2, PBRM1*, and *NEK4* genes, and upregulation of *TMEM110*. By contrast, the locus marked by rs1108842 down-regulates the *GNL3* and *NEK4* genes while upregulating *PBRM1* and *TMEM110*. Finally, the locus marked by rs2710323 down-regulates *NEK4* but upregulates the other genes.

### Common variant association signals from different ethnicities show extensive connectivity

SNPs within the *IGF2BP2* gene that were previously identified, by a transancestral GWAS metaanalysis (Horikoshi et al., 2016), as being common in East Asian, European, South Asian, African American, and Mexican American populations were analyzed to determine the pattern of connectedness for these SNPs. The transancestral SNPs were located within a 52,598 bp region spanning the terminal 5′ intron of *IGF2BP2* and were involved in cis-eQTLs with *IG2FBP2* itself (Supplementary Figure 4). Notably, the trans-acting SNPs (rs13100823, rs11705729, and rs11927381) are in strong LD (>0.97 *r*^2^). However, they are located on different MboI restriction fragments (Supplementary Figure 4) and act as eQTLs in a gene and tissue specific manner (Table 4). Trans-acting eQTL SNPs associated with the *IGF2BP2* locus were also observed between rs4402960—*GRM1* (Chr 6), rs1470579—*CCDC14* (Chr 3), and rs1470579—*SND1* (Chr 7).

**Table 4:** Three significant (FDR ≤ 0·05) tissue-specific trans-interactions were identified between eQTL SNPs within the fine-mapped *IGF2BP2* region on chromosome and three genes on different chromosomes.

**eQTL SNP**	**Gene**			**GTEx eQTL**		**q-value**	Cell line^*^
	**Name**	**Chr**	**Start (bp)**	**Tissue^¥^**	***p*-value (E-05)**		
rs13100823	*RBM47*	4	40,425,272	Whole_Blood	1.68	0.049	HUVEC
rs11705729	*KIAA1430*	4	186,080,819	Hypothalamus	1.18	0.036	NHEK
rs11927381	*DIS3L2*	2	232,825,955	Lung	1.08	0.034	NHEK

Thirty-three out of the 36 fine-mapped SNPs within the IGF2BP2 region were identified as significant cis-acting eQTLs within the thyroid tissue. Trans eQTL SNPs were defined as occurring between loci > 1Mb apart, or on different chromosomes, with a FDR ≤ 0.05.

* Cell line in which the SNP-gene interaction was captured.

¥ *Tissue the eQTL was identified in*.

### Comorbidity: pathway interactions or shared genes?

Comorbidity can be explained by direct effects on the same genes or epistatic effects acting through pathways. Notably, only sixteen common genes (Supplementary Table 3) were affected by eQTL SNPs that were linked to obesity and type 2 diabetes (Supplementary Table 4). Of these, only *ARAP1* and *BRD7* were affected by globally significant SNPs (i.e., rs8050136 and rs11603334) that had previously been associated with both obesity and type 2 diabetes.

**Figure 2:**
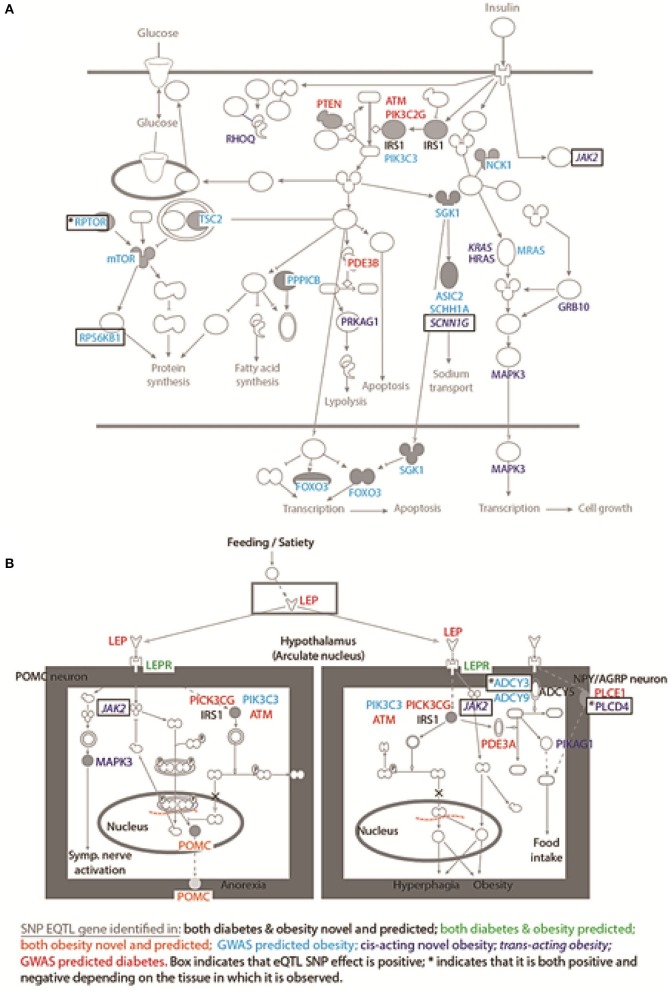
SNPs mark regulatory regions that act to regulate genes within the glucose-insulin and leptin signaling pathways. Novel and predicted eQTL SNP-gene interactions fall within: **(A)** the glucose-insulin; and **(B)** the leptin signaling pathways. The dominant effect for the eQTL SNPs is to down-regulate the gene transcript level, consistent with the SNP falling within an enhancer region. Novel eQTL SNP-gene pairs contribute numerous regulatory interactions to both pathways including: trans-regulatory connections (e.g.*, JAK2*); and combined action on single genes (i.e.*, IRS1 and POMC*).

**Figure 3:**
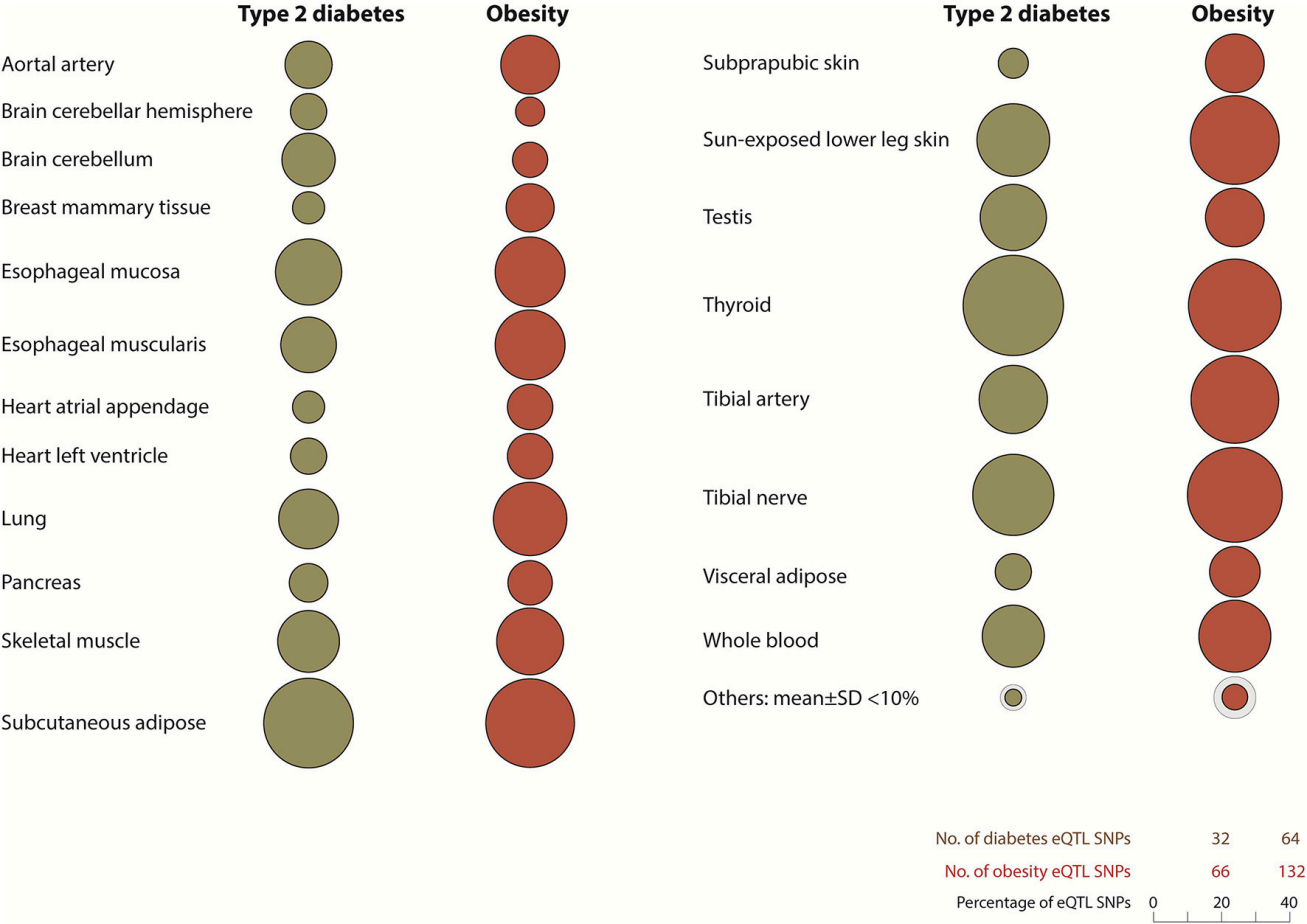
Diabetes and obesity disease-associated spatial SNPs with significant eQTL effects (*FDR* > 0.05) are unevenly distributed throughout human tissues. Tissues with <10% total number of spatial eQTL SNPs in type 2 diabetes and obesity include the liver (1.3, 3.9%), stomach (7.5, 9.7%), and pituitary gland (8.1, 8.2%) respectively. Other tissues: adrenal gland, atrial aorta, coronary artery, brain - anterior cingulate cortex (BA24), brain - caudate basal ganglia, brain - cortex, brain - frontal cortex (BA9), brain - hippocampus, brain - hypothalamus, brain - nucleus accumbens basal ganglia, brain - putamen basal ganglia, breast - mammary tissue, sigmoid colon, transverse colon, gastroesophageal junction, liver, ovary, pituitary, prostrate, spleen, stomach, testis, uterus, and vagina. All eQTL SNP-genes included in this analysis were expressed with an RPKM of >1.0 (GTEx version 4.1, accessed on 09/30/16).

eQTL SNP-gene pairs for obesity and type 2 diabetes showed evidence for significant epistatic interactions within the glucose-insulin and leptin signaling pathways (Figure 2). However, the number and distribution of significant eQTL effects (FDR < 0.05) associated with the SNP-gene pairs occurred in a disease and tissue specific manner (Figure 3 and Supplementary Figure 5). The observed tissue specific distribution of the eQTL SNP-gene pairs for the non-diabetes associated SNPs was significantly different to that obtained for the diabetes associated SNPs for all tissues (*t*-test *p*-value = 0·001347; Supplementary Figure 6). Restricting the effect to eQTL SNP-gene pairs in which the gene was expressed at >1.0 Read(s) Per Kilobase of transcript per Million mapped reads (RPKM), to reduce the impact of very lowly expressed genes, identified some small differences in the disease and tissue specific distributions of the effects (compare Figure 3 and Supplementary Figure 5).

The disease and tissue specificity of the spatial eQTL SNP-gene pairs can be further classified according to the metabolic function(s) that the interacting gene is involved in (Supplementary Table 5). Analysis using the curated IPA knowledgebase identified enrichment for genes involved in lipid metabolism in the following tissues: adipose (*p* < 3.07 × 10^−2^), skeletal muscle (*p* < 1.57 × 10^−2^), and pancreas (*p* < 4.93 × 10^−2^; Supplementary Spreadsheet 3 doi: 10.17608/k6.auckland.5285044). Notably, there was no enrichment for eQTL SNP-gene pairs involving genes for lipid metabolism within the liver.

SNPs associated with fasting insulin-based measures of insulin resistance have previously been linked to a reduction in subcutaneous adipose tissue and adverse metabolic profiles (Yaghootkar et al., 2014). Re-analysis of these SNPs, using our approach, revealed that they mark loci that spatially regulate genes in tissues central to metabolism including subcutaneous adipose, visceral adipose, and thyroid (Supplementary Table 6). The strength of our integrative approach is again highlighted as 14 of the spatially regulated genes were not previously associated with the SNPs but may contribute to the mechanistic interpretation of metabolic dysfunction e.g., *PPA2*, a negative regulator of the insulin metabolic signaling pathway and *CCTN2*, a regulator of leptin (Ugi et al., 2004) and *ADIPOQ* (Broholm et al., 2016).

## Discussion

Here we identify the functional effects of loci marked by SNPs associated with diabetes and/or obesity. Our results identify differential regulation of genes by loci marked by diabetes and obesity associated spatial eQTL SNPs. These regulatory interactions occur in a disease and tissue specific manner. We identify sets of eQTLs in tissues that are known to be involved in energy homeostasis and adiposity (e.g., thyroid Milionis and Milionis, 2013 and subcutaneous adipose Lee et al., 2013). We also identify sets of eQTLs in tissues that are not classically associated with diabetes or obesity. Our findings are consistent with the diabetes and obesity associated genetic variants acting in an individual, tissue, and developmental stage-specific manner.

The identification of SNP-gene pairs is central to our approach to integrate these orthogonal data sets. To do this we rely upon high-resolution (i.e., 1 kb) Hi-C data captured from eight non-synchronized immortalized human cell lines (Supplementary Table 1). It can be argued that the identification of these SNP-gene interactions should incorporate a measure of the significance of the Hi-C data. However, there is: (a) a high level of conservation of topologically associated domains between cell lines and lineages (Dixon et al., 2015); and (b) Hi-C contacts captured from a population of cells represent a stochastic structure in which permissible interactions occur at a detectable frequency even when they are not the dominant interactions (Nagano et al., 2013). Thus, we contend that the interactions that we used represent those that are capable of forming within the human genome. Despite this, it is clear that trans interactions show much greater cell-type specificity. Therefore, future work should incorporate tissue and developmental stage specific Hi-C maps into the analysis to ensure the complete identification of all possible SNP-gene pairs.

The eQTL SNP-gene connections we described were identified across and not within a single population. This complication arose because the Hi-C cell lines and GTEx data we used were not generated from the same samples. While this may be suboptimal, we contend that previous transferability studies have identified common genetic variants that have regular effects across populations (Waters et al., 2010; Saxena and Palmer, 2016). Consistent with this, we identified a series of regulatory connections that involved previously identified fine-mapped transancestral SNPs within intron 1 of the *IGF2BP2* locus (Horikoshi et al., 2016). In addition to strong zones of cis-regulation of *IGF2BP2* itself, three of the transancestral eQTL SNPs within intron 1 of *IGF2BP2* regulate diabetes and obesity related genes in trans (i.e., *RBM47* Benton et al., 2015, *KIAA1430* Sandholm et al., 2017, and *DIS3L2* Kim et al., 2010). Notably, the cis eQTLs affect the thyroid, while trans eQTLs affect the lung, hypothalamus and whole blood tissues. Despite the fact that there is no Hi-C data that identifies race specific changes in genome organization, the identification of effects associated with transancestral SNPs is consistent with allele frequencies impacting on elements responsible for the formation, maintenance, or regulatory outcomes of SNP-gene interactions. Therefore, allele frequency dependent changes to tissue specific eQTL distributions may contribute to race specific differences in the development and progression of diabetes and obesity. As such, it is important to match samples and integrate the Minor Allele Frequencies (MAFs) of the variants (Auton et al., 2015) into future investigations.

The etiological association of diabetes and obesity spatial eQTLs can follow any of the four described models for comorbidity pathways (Valderas et al., 2009): (1) the direct causation model where the genetic variants for one disease directly cause the second disease; (2) the association model, in which the genetic variants for the two diseases are correlated and thus increase the likelihood of the diseases occurring simultaneously; (3) the heterogeneity model, whereby the genetic variants are uncorrelated but each can cause the comorbid diseases; or (4) the independence model, in which the comorbid diseases arise as a result of a third distinct disease condition. We observed a low direct overlap between the eQTLs for type 2 diabetes and obesity. Yet, regions marked by obesity and type 2 diabetes SNPs were associated with numerous significant regulatory impacts on genes within the glucose-insulin and leptin signaling pathways. These observations are consistent with the rewiring of physical and genetic interaction networks across complex disease conditions (Muoio and Newgard, 2008; Hou et al., 2014; Hu et al., 2016; Boyle et al., 2017). Collectively our results indicate that the comorbidity pathway for diabetes and obesity is likely due to the combined effect of correlated changes within pathways and tissues.

The tissue specificity of the regulatory effects we identified is consistent with current observations of the dynamic changes that occur within the local and global landscapes of genome organization in different cell types (Dixon et al., 2015; Doynova et al., 2016). The partitioning we observed for the diabetes and obesity associated SNPs was significantly different to the tissue-specific profiles for non-diabetes associated SNPs (*p* < 0.001). This finding reinforces the high discovery potential of integrating diverse sets of partially orthogonal data. This is particularly pertinent for polygenic type 2 diabetes and obesity where the metabolic dysbiosis is associated with a fundamental breakdown in the ability of the body to maintain and regulate a stable equilibrium for energy metabolism. The increased numbers of obesity eQTLs may reflect the fact that obesity can result from perturbations to a greater number of pathways, or at more points within these pathways, than diabetes. However, the absolute number of eQTL SNP-gene pairs that are influencing gene regulation in a particular tissue is not necessarily a direct measure of the significance of the changes for either diabetes or obesity.

We undertook a discovery approach that makes no *a priori* assumption of tissue relevance. This was necessary because the GTEx database is a growing resource that does not currently include all the tissues that are classically considered “relevant” to the pathogenesis of Type 2 diabetes or obesity. The utility of our discovery-based approach is reinforced by studies on Huntington's disease that have identified pre-pathology changes in tissues, which were not previously associated with the pathogenesis or progression of Huntington's disease (Carroll et al., 2015). Treatment of these tissue specific changes are therapeutically possible, can delay onset and improve quality of life for Huntington's carriers. Therefore, the incorporation of tissues that are not classically associated with diabetes or obesity into our analysis potentially informs on system-wide changes that contribute to the development of the disorders. Future expansion to cell-type specific data that overcomes mRNA averaging effects is important. However, these approaches also suffer from limitations caused by the artificial nature of the environment, lack of appropriate cell-to-cell communication, and tissue manipulation etc. Future work aimed at understanding how SNPs contribute to these disorders through specific pathological pathways, for example impacts on the insulin secreting islets that constitute <1% of the pancreas, will require an approach that integrates cellular and tissue specific understandings.

**Figure 4:**
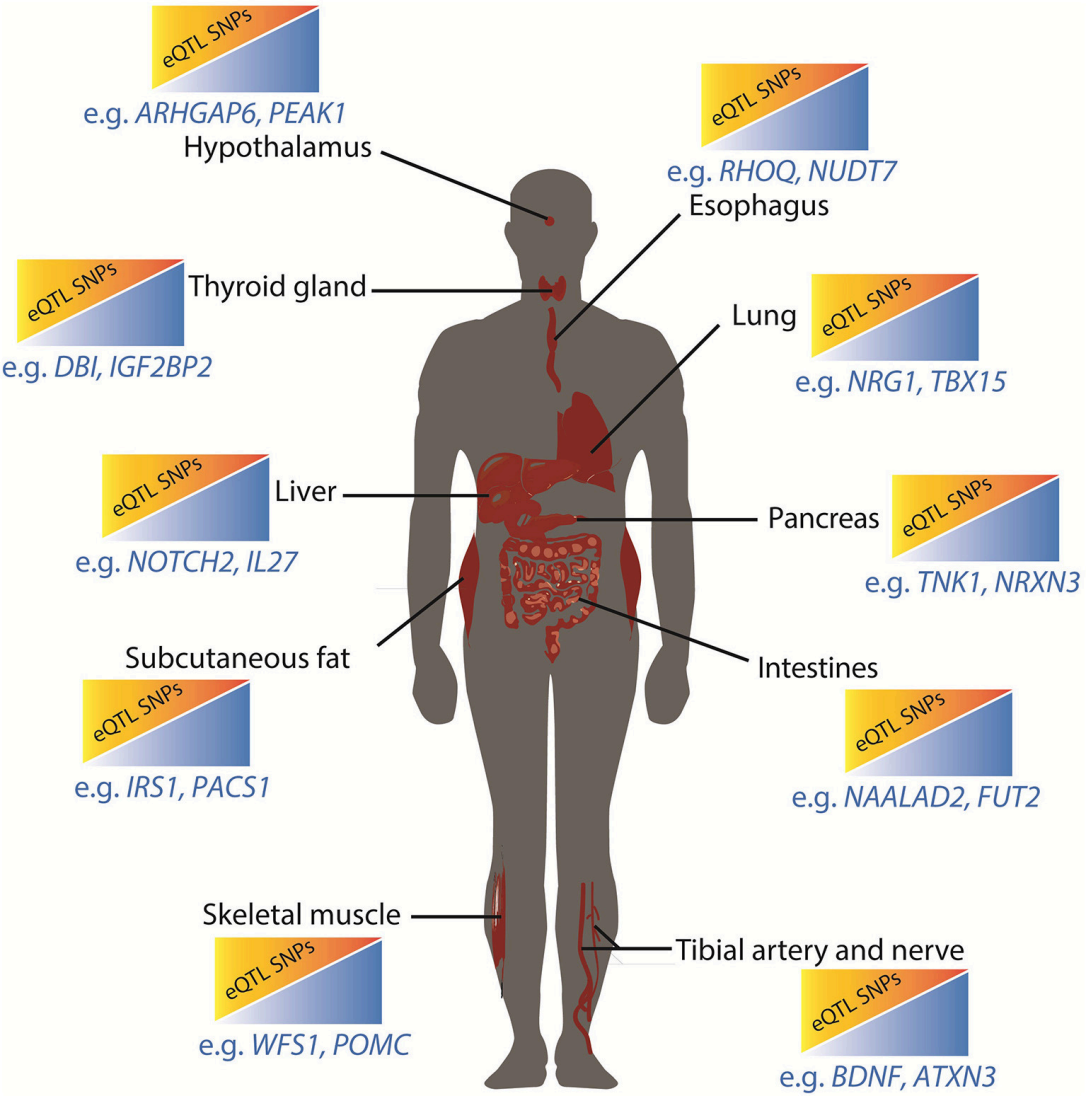
Metabolic restriction model for integrated effects of diabetes and obesity associated SNPs. In this model, increasing the number of obesity and type 2 diabetes associated eQTL SNP-gene interactions in critical tissues results in small but cumulative increases in risk due to reductions in capacity to respond to peak energy supply. Genes that are subject to tissue specific eQTL effects are annotated. The esophagus, lungs, and tibial artery and nerve do not have established roles in the regulation of metabolic functions although there are associations between these organs (or their dysfunction) and diabetes and obesity.

Our results provide novel insights into the separate roles of different adipose repositories in the development of the metabolic syndrome (Lee et al., 2013; Neeland et al., 2013; Liesenfeld et al., 2015; Tahara et al., 2015). For example, the adipose expandability hypothesis attempts to explain the well-known differential effects of subcutaneous and visceral fat on diabetes and obesity. This hypothesis posits that the capacity of subcutaneous adipose to store fat and modulate circulating adipokines can be exceeded, after which adipokine derangements and ectopic fat deposition occur (Tan and Vidal-Puig, 2008; Lagathu et al., 2010; Cuthbertson et al., 2017). Thus, our observation that subsets of population level diabetes and obesity associated SNPs impact on gene expression within the subcutaneous fat, potentially altering its capacity to store fat and moderate levels of circulating adipokines, is notable. Specific individuals contain different combinations of SNPs. Thus, it is possible that genotype specific combinations of diabetes and obesity associated SNPs, which mark regulatory regions that have negative effects on lipid related gene expression within subcutaneous fat, can partially explain individual responses. Unlike single gene disorders, loss of regulatory responsiveness need not be catastrophic but may lead to a small but compounding increased risk over an individual's lifetime. eQTL SNP-gene interactions occurring in other tissues critical to metabolic control (e.g., pancreatic cells; Figure 4) can increase this risk further.

In conclusion, we propose that the identity and number of obesity and diabetes spatial eQTL SNP-gene pairs that are active within different tissues reduces the ability of these tissues to contribute to the maintenance of a healthy energy metabolism (Figure 4). Environmental conditions, including absolute levels of food and exercise, can moderate this genetic risk. Thus, the clinical risk for polygenic disorders is the sum result of the gene-environment interactions that occur within the context of a four-dimensional regulatory network that is “tuned” by the combinatorial action of regulatory sites that spatially cluster and are subject to genetic variation. Future personalized studies that integrate an individual's tissue specific eQTL profile with longitudinal measurements of clinical biomarkers of type 2 diabetes and obesity will clarify the different mechanisms that contribute to the development and apparent paradoxes that are associated with these disorders.

## Author contributions

CE wrote CoDeS3D. TF ran analyses, contributed to the software, interpreted data and co-wrote the manuscript. JI participated in discussions and commented on the manuscript, WS contributed to data interpretation and commented on the manuscript, JO directed the study, contributed to data interpretation and co-wrote the manuscript. JO is guarantor for this article.

### Conflict of interest statement

The authors declare that the research was conducted in the absence of any commercial or financial relationships that could be construed as a potential conflict of interest.
