# Supplementary material for: Physical Interactions and Expression Quantitative Traits Loci Identify Regulatory Connections for Obesity and Type 2 Diabetes Associated SNPs
Source: Front Genet. 2017 Oct 13;8:150. doi: 10.3389/fgene.2017.00150 (PMC5645506; doi:10.3389/fgene.2017.00150)
Supplement: Supplementary file 1 [file DataSheet1.docx]

Supplementary Material

Regulatory Links Between Obesity and Type 2 Diabetes Revealed using Hi-C Data and Expression Quantitative Traits Loci.

Tayaza Fadason^1ǂ^, Cameron Ekblad^1ǂ^, John R. Ingram^2^, William Schierding^1^, Justin M. O’Sullivan^1*^

^1^ Liggins Institute, The University of Auckland, Auckland, New Zealand

^2^ The New Zealand Institute of Plant and Food Research, Mount Albert, Auckland, New Zealand

^ǂ^ equal contribution

*corresponding author: Justin M. O’Sullivan, The Liggins Institute, The University of Auckland, Phone:+64 9 9239868, [justin.osullivan@auckland.ac.nz](mailto:justin.osullivan@auckland.ac.nz)

# Supplementary Methods

Source code for the pipeline Contextualising Developmental SNPs in Three Dimensions (CoDeS3D) is available at <https://github.com/alcamerone/codes3d>

# Supplementary Data

2.1 Supplementary Spreadsheet 1. Detailed data of obesity spatial eQTL-gene interactions (doi:10.17608/k6.auckland.5285038)

2.2 Supplementary Spreadsheet 2. Detailed data of type 2 diabetes spatial eQTL-gene interactions (doi:10.17608/k6.auckland.5285041)

2.3. Supplementary Spreadsheet 3. Effects of spatial obesity and type 2 diabetes eQTLs in subcutaneous adipose, visceral adipose, skeletal muscle, liver and pancreas 3 (doi:10.17608/k6.auckland.5285044)

# Supplementary Figures and Tables

## Supplementary Figures


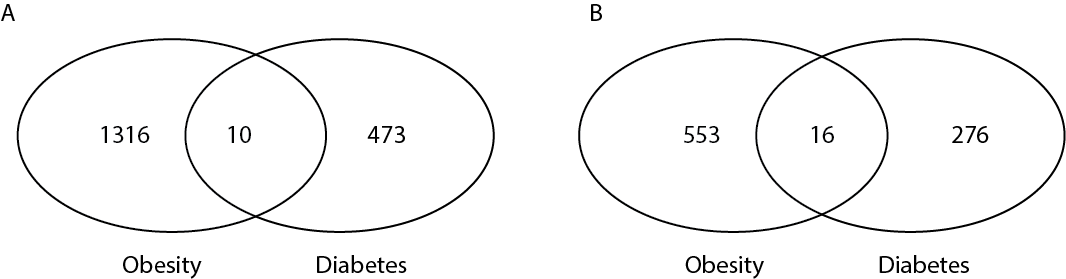


**Supplementary Figure 1**. A: Ten SNPs that are associated with diabetes and obesity, at p values that are suggestive of global significance (p≤9·0 x 10^-6^) (Supplementary Table 2), are shared between both type 2 diabetes and obesity in the GWAS database. B: CoDeS3D identified sixteen genes that are affected by eQTL SNPs that are associated with both diabetes and obesity. Fourteen of these genes are regulated by eQTL SNPs that are not shared between diabetes and obesity (Supplementary Table 3).


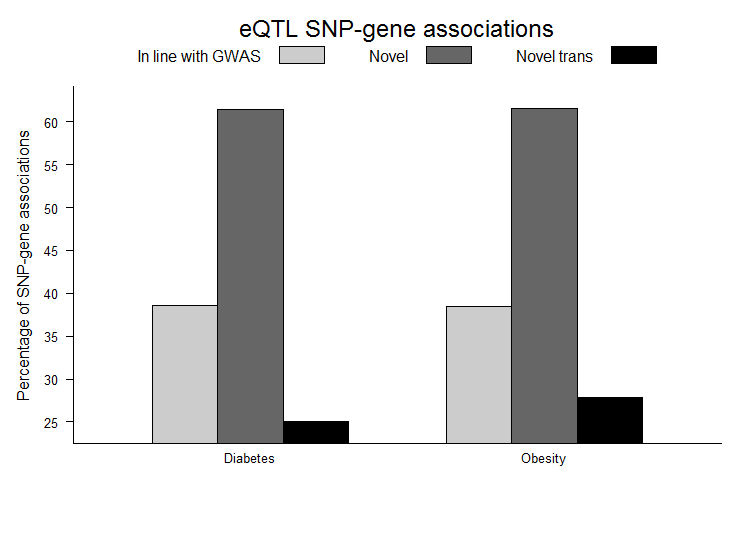


Supplementary Figure 2. More than 60% of the eQTL SNP associations that were identified in GTEx were novel and were not associated with the diseases in the GWAS database. Of the novel eQTL SNP-gene pairs that were identified for diabetes and obesity (Supplementary Spreadsheets 1 & 2), 24·9% and 28·0%were trans-acting and regulated genes > 1 Mb away or on another chromosome to the SNP.


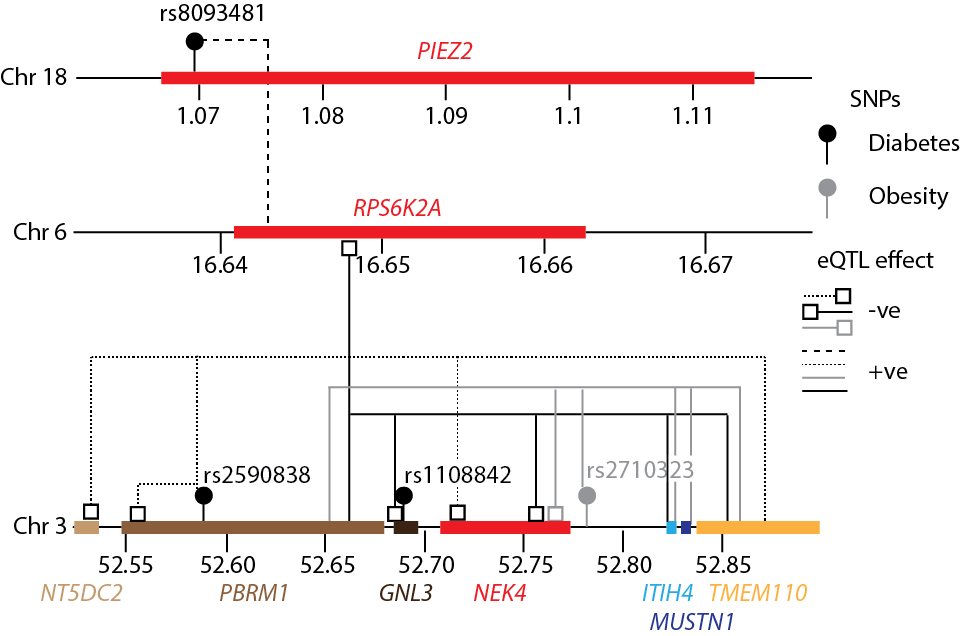


**Supplementary Figure 3.** Co-regulation of genes by spatial obesity and type 2 diabetes eQTL SNPs. SNPs rs2590838, rs1108842 and rs2710323 are in high LD (0·78 – 0·8) however their effects are gene specific . The different line strokes distinguish different SNP-gene interactions. Notably, rs1108842 within the *GNL3* gene on chromosome 3 trans-regulates *RPS6KA2* on chromosome 6, which is also regulated by another trans interaction with rs8093481 on chromosome 18. There was only one HiC interaction captured supporting the connection between the MboI fragments containing rs1108842 and *RPS6KA2* in the cell lines used in this study. Intronic variants of *RPS6KA2* have been associated with diabetic cataracts in the Taiwanese population. Moreover, epigenetic modifications within *RPS6KA2* have been linked to proliferative diabetic retinopathy, and the gene product of *RPS6KA2* is linked to insulin resistance through the mTOR signaling pathway (<http://www.kegg.jp/kegg-bin/show_pathway?hsa04931+6196)>.


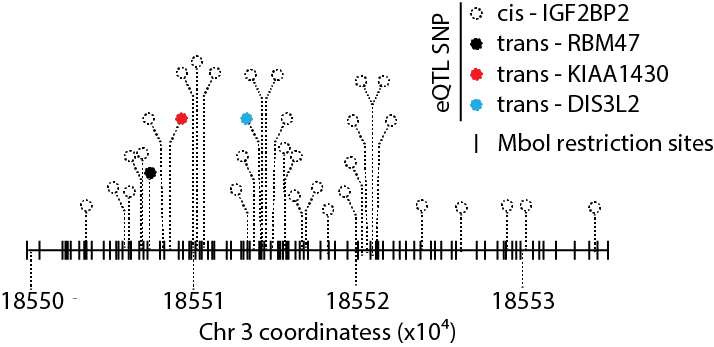


**Supplementary Figure 4**. Trans-ancestral SNPs within *IGFBP2* have cis and trans-regulatory effects. The trans acting SNPs rs13100823, rs11705729, and rs11927381 are in LD but act as eQTLs for different genes in a tissue specific manner. This observation is consistent with the SNPs being located on different MboI restriction fragments, which are captured interacting with different genomic regions.


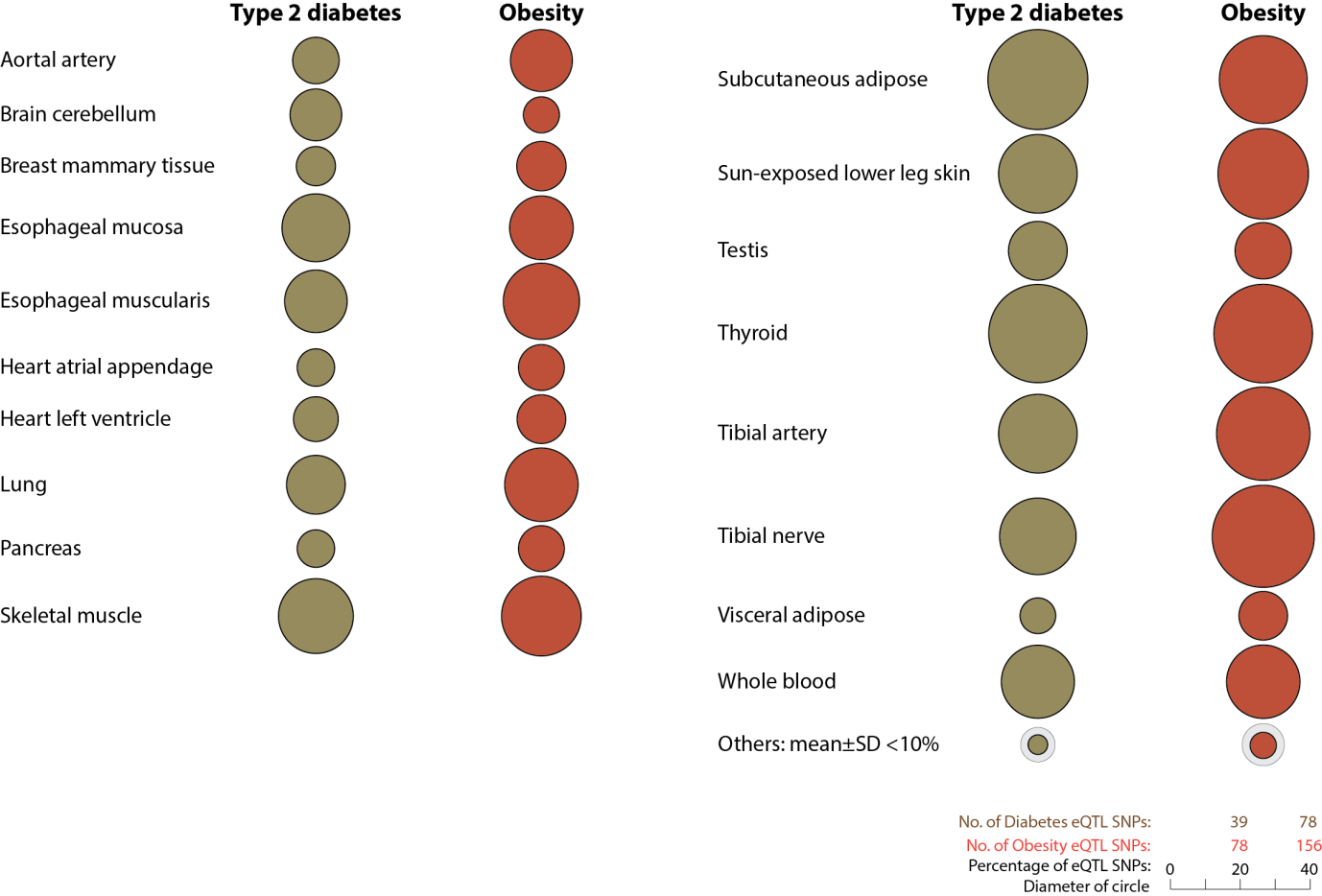


**Supplementary Figure 5.** The number of disease-associated spatial SNPs with significant eQTL effects (*FDR* > 0·05) varies across tissues. Tissues with <10% total number of spatial eQTL SNPs in type 2 diabetes and obesity include the liver (2·6%, 4·4%), stomach (7·1%, 9·2%) and pituitary gland (7·7%, 8·0%) respectively. Other tissues: adrenal gland, atrial aorta, coronary artery, brain - anterior cingulate cortex (BA24), brain - caudate basal ganglia, brain - cerebellar hemisphere, brain - cortex, brain - frontal cortex (BA9), brain - hippocampus, brain - hypothalamus, brain - nucleus accumbens basal ganglia, brain - putamen basal ganglia, sigmoid colon, transverse colon, gastroesophageal junction, liver, ovary, pituitary, prostrate, spleen, stomach, uterus and vagina. Spatial eQTL SNPs are included here regardless of the expression levels (RPKM) of the genes they regulate. The distribution of eQTL-SNP gene pairs across human tissues was significantly different (p<0·0001, t-test for correlated samples) for GWAS SNPs associated with diabetes and obesity (p<9 x 10^-6^). eQTL data was obtained from the GTEx Portal (Version 4·1, 09/30/16) for spatially associated SNP-gene pairs.


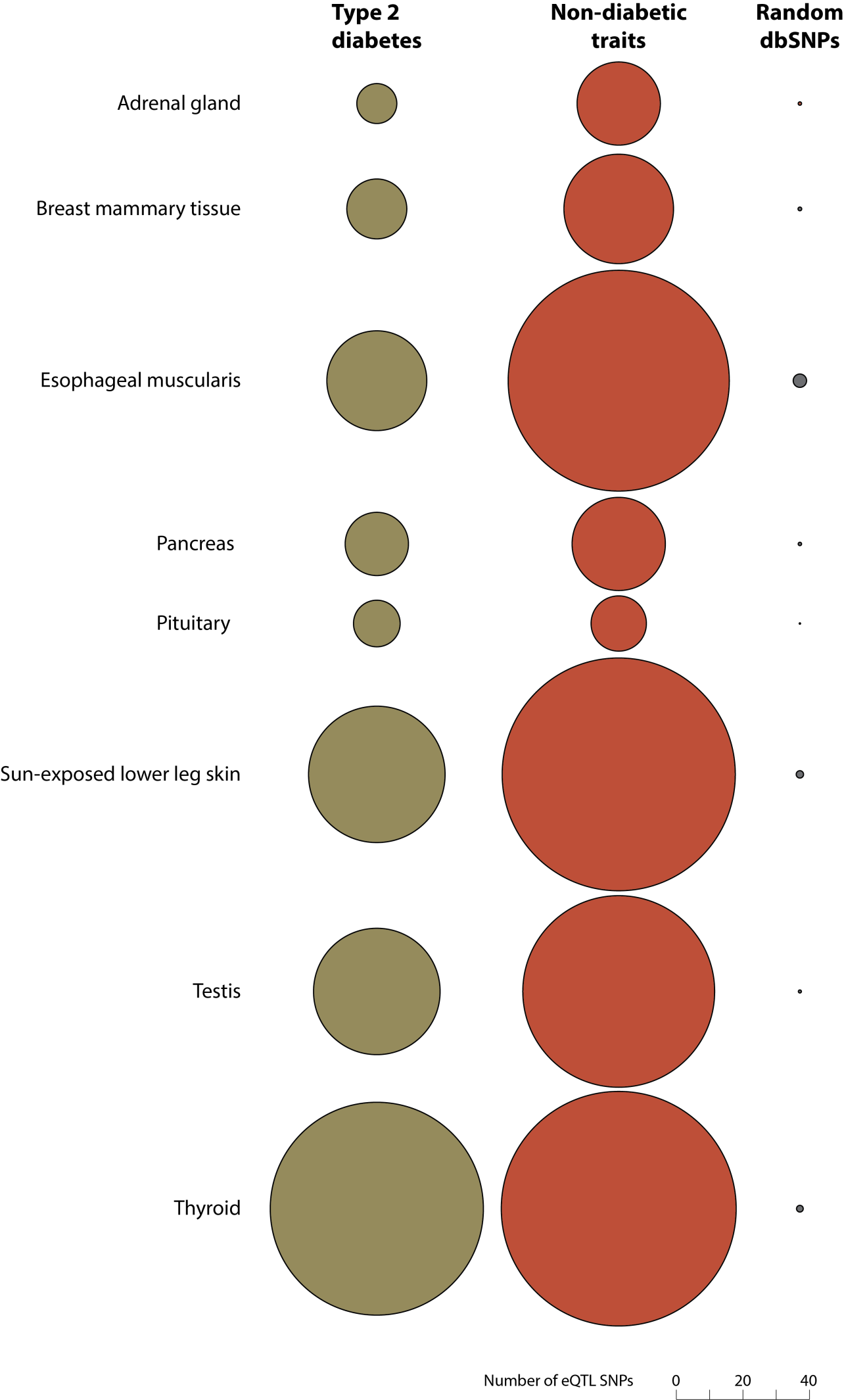


**Supplementary Figure 6.** To control for the tissue distribution observed in type 2 diabetes, we used the Monte Carlo method on 1000 sets of randomly chosen 483 SNPs from dbSNP (Table 2) and 57 non-diabetic related traits from the GWAS Catalog (Supplementary Table 5) to test for spatial eQTL interactions and their tissue distribution (as means of total eQTL-SNP gene pairs). The patterns of eQTL interactions observed in all tissues of the dbSNP and non-diabetic controls were significantly different (p-values of <0.00001 and 0·001347 respectively) to tissues in type 2 diabetes.

## Supplementary Tables

**Supplementary Table 1.** Data sources used in this study.

| Data | Notes | Access | Reference |
| --- | --- | --- | --- |
| High resolution Hi-C data | GM12878  IMR90 (CCL-186)  HMEC (CC-2551)  NHEK (192627)  K562 (CCL-243)  KBM7  HUVEC  HeLa | Gene Expression Omnibus (GSE63525) | ^1^ |
| eQTL data | GTEx version multi-tissue eQTLs analysis v4. | GTEx portal  (http://www.gtexportal.org/home/) |  |

**Supplementary Table 2.** SNPs associated with non-diabetes associated phenotypes (GWAS Catalog; p<10^-6^) were used as negative controls for tissue distribution (Supplementary Figure 6). The table lists the phenotypes that were used to source the non-diabetes SNPs.

| GWAS Catalog traits | | |
| --- | --- | --- |
| Acute lymphoblastic, leukemia  AIDS  Alcohol and nicotine codependence  Alcohol dependence  Alpha wave measurement, electroencephalogram measurement  Antibody measurement, Epstein-Barr virus infection  Astigmatism  Basal cell carcinoma  Brain aneurysm  Brain connectivity measurement  Cleft lip  Cleft palate, cleft lip  Corneal topography  Creutzfeldt Jacob Disease  Dental caries  DNA methylation  Ear protrusion  Eye color  Eye measurement  freckles  Freckles, sunburn | Gene methylation measurement  Hair color  Hair morphology  HIV viral set point measurement  HIV-1 infection  HIV-1 infection, bilirubin measurement  HIV-1 infection, body fat distribution  HIV-1 infection, high density lipoprotein cholesterol measurement  HIV-1 infection, response to efavirenz, virologic response measurement  HIV-1 infection, Susceptibility to viral and mycobacterial infections  HIV-1 infection, virologic response measurement, response to abacavir  Hodgkins lymphoma  HPV seropositivity  Intra cranial volume  Leprosy  Malaria  Melanoma | Neuroblastoma  Orofacial clefting syndrome  Prion disease  Prion disease, mood disorder  Reading and spelling ability  reasoning  Recombination rate  Sickle cell anemia  Smooth surface dental caries  Social communication impairment  sunburn  Suntan  Telomere length  Tooth eruption  Tuberculosis  Typhoid fever  Word list delayed recall measurement, memory performance  Word list delayed recall measurement, memory performance, paragraph delayed recall measurement  Word reading |

**Supplementary Table 3.** There were sixteen genes that were subject to regulation by eQTL SNPs in both obesity and diabetes.

| Gene | Disease^†^ | Interaction | SNP | HiC Cell Lines ^¥^ | eQTL Tissue^ǂ^ |
| --- | --- | --- | --- | --- | --- |
| *ABO* | Obesity | Cis | rs657152 | HUVEC,NHEK,IMR90,KBM7 | Thyroid |
|  | T2D | Cis | rs651007 | HUVEC,IMR90,NHEK,KBM7 | Whole_Blood |
| *ARAP1* | Obesity | Cis | rs11603334 | HUVEC,NHEK,K562,HMEC,IMR90,KBM7 | Esophagus_Mucosa |
|  | T2D | Cis  Cis | rs11603334  rs1552224 | HUVEC,NHEK,K562,HMEC,IMR90,KBM7  HUVEC,NHEK,K562,HMEC,IMR90,KBM7 | Esophagus_Mucosa  Esophagus_Mucosa |
| *BDR7* | Obesity | Trans | rs8050136 | K562 | Pituitary |
|  | T2D | Trans | rs8050136 | K562 | Pituitary |
| *CRYL1* | Obesity | Cis | rs4770049 | HUVEC,NHEK,K562,HMEC,IMR90,KBM7 | Adipose_Subcutaneous |
|  | T2D | Trans | rs17376456 | HUVEC | Pituitary |
| *IRS1* | Obesity | Cis | rs2176040 | HUVEC | Adipose_Subcutaneous |
|  | T2D | Cis  Cis  Cis  Cis | rs1515110  rs2943641  rs2943640  rs925735 | HUVEC,IMR90,NHEK,KBM7  HUVEC  NHEK  K562,HUVEC,NHEK,KBM7 | Adipose_Subcutaneous  Adipose_Subcutaneous  Adipose_Subcutaneous  Adipose_Subcutaneous |
| *ITIH4* | Obesity | Cis | rs2710323 | K562,HUVEC,NHEK,IMR90 | Whole_Blood |
|  | T2D | Cis | rs1108842 | KBM7 | Whole_Blood |
| *KCNJ11* | Obesity | Cis | rs1557765 | K562,HUVEC,NHEK,IMR90,KBM7 | Skin_Sun_Exposed_Lower_leg  Esophagus_Mucosa  Skin_Not_Sun_Exposed_Suprapubic |
|  | T2D | Cis  Cis | rs5215  rs5219 | K562,HUVEC,IMR90,NHEK,KBM7  K562,HUVEC,IMR90,NHEK,KBM7 | Skin_Not_Sun_Exposed_Suprapubic  Skin_Not_Sun_Exposed_Suprapubic |
| *METTL10* | Obesity | Cis | rs3740540 | KBM7 | Thyroid |
|  | T2D | Trans | rs1802295 | NHEK | Thyroid |
| *NCR3LG1* | Obesity | Cis | rs1557765 | HUVEC,NHEK,IMR90,KBM7 | Esophagus_Mucosa  Nerve_Tibial  Breast_Mammary_Tissue  Skin_Sun_Exposed_Lower_leg  Skin_Not_Sun_Exposed_Suprapubic  Artery_Tibial  Thyroid  Cells_Transformed_fibroblasts |
|  | T2D | Cis  Cis | rs5215  rs5219 | K562,HUVEC,IMR90,NHEK,KBM7  K562,HUVEC,NHEK,KBM7 | Thyroid  Thyroid |
| *NEK4* | Obesity | Cis | rs2710323 | K562,NHEK,IMR90,KBM7 | Cells_Transformed_fibroblasts  Artery_Tibial  Thyroid  Nerve_Tibial  Skin_Sun_Exposed_Lower_leg  Esophagus_Mucosa  Adipose_Visceral_Omentum  Muscle_Skeletal  Heart_Atrial_Appendage  Breast_Mammary_Tissue  Esophagus_Muscularis |
|  | T2D | Cis  Cis | rs1108842  rs2590838 | HUVEC,NHEK  HUVEC,NHEK | Thyroid  Thyroid |
| *PPARG* | Obesity | Cis | rs11709077 | NHEK | Heart_Left_Ventricle |
|  | T2D | Cis | rs1801282 | HUVEC,NHEK,K562,HMEC,IMR90,GM12878,KBM7 | Heart_Left_Ventricle |
| *SLC38A11* | Obesity | Cis | rs355810 | HUVEC,NHEK | Muscle_Skeletal |
|  | T2D | Cis | rs3923113 | IMR90,NHEK | Breast_Mammary_Tissue |
| *SYN2* | Obesity | Cis | rs11709077 | HUVEC | Muscle_Skeletal |
|  | T2D | Cis  Cis | rs17036101  rs1801282  rs13081389 | HUVEC,NHEK,K562,IMR90,GM12878,KBM7  HUVEC,NHEK,K562,HMEC,GM12878,KBM7  K562,HUVEC,IMR90,NHEK,KBM7 | Heart_Atrial_Appendage  Muscle_Skeletal  Esophagus_Muscularis |
| *TH* | Obesity | Cis | rs7111341 | NHEK | Skin_Not_Sun_Exposed_Suprapubic |
|  | T2D | Cis | rs11043007 | HUVEC,IMR90 | Skin_Not_Sun_Exposed_Suprapubic |
| *TMEM110* | Obesity | Cis | rs2710323 | HMEC,HUVEC,NHEK,IMR90,KBM7 | Artery_Aorta  Adipose_Subcutaneous  Muscle_Skeletal  Cells_Transformed_fibroblasts  Lung  Whole_Blood |
|  | T2D | Cis | rs1108842  rs2590838 | IMR90,NHEK  IMR90,NHEK,KBM7 | Whole_Blood  Whole_Blood |
| *UGGT2* | Obesity | Cis  Cis | rs7989336  rs9634489 | NHEK,KBM7  NHEK,IMR90 | Skin_Not_Sun_Exposed_Suprapubic  Pancreas |
|  | T2D | Cis | rs2038823 | IMR90,KBM7 | Skin_Not_Sun_Exposed_Suprapubic |

^†^, NHGRI-EBI GWAS Catalog version v1.1 downloaded 13/07/2016 **^¥^**, Rao *et al*. 2014; **^ǂ^,**GTEX version 4**·**1 accessed 09/30/16

**Supplementary Table 4.** SNPS that have been associated with both diabetes and obesity GWAS with a significance level p<0·5 x 10^-8^.

| SNP | Context | Trait | Mapped Genes |
| --- | --- | --- | --- |
| rs9939609 | Intron variant | Type 2 diabetes, Body mass index | *FTO* |
| rs7903146 | Intron variant | Type 2 diabetes, Metabolic syndrome, Proinsulin levels,  Body mass index | *TCF7L2* |
| rs998584 | Downstream gene variant | Adiponectin levels, Body mass index | *VEGFA - LOC105375070* |
| rs16862964 | Intergenic variant | Type 2 diabetes (young onset) and obesity | *LOC105374268* |
| rs715 | 3’ UTR variant | Metabolite levels, Body mass index | *CPS1* |
| rs8050136 | Intron variant | Type 2 diabetes, Adiposity, Weight | *FTO* |
| rs12970134 | Intergenic variant | Type 2 diabetes, Weight | *LOC105372154 - LOC105372155* |
| rs10830963 | Intron variant | Type 2 diabetes, Fasting glucose-related traits, Glucose homeostasis traits, Obesity-related traits | *MTNR1B* |
| rs11603334 | 5’ UTR variant | Proinsulin levels, Body mass index | *ARAP1* |
| rs6595551 | Downstream gene variant | Type 2 diabetes (young onset) and obesity | *LOC105379156 - LOC105379157* |

**Supplementary Table 5.** Top canonical pathways and biological functions of eGenes identified by IPA in pancreas, adipose, liver, brain, skeletal muscle and thyroid.

| Canonical pathways | Tissues | N^o·^ eGenes | P value | Biological functions | Tissues | N^o·^ eGenes | P value range |
| --- | --- | --- | --- | --- | --- | --- | --- |
| Arsenate detoxification I | P | 1/4 | 1·34E-02 | Antigen presentation | L | 1 | 5·95E-03 – 1·70-03 |
| D-myo-inositol (1,4,5)-triphosphate biosynthesis | L | 1/27 | 2·28E-02 | Carbohydrate metabolism | T | 15 | 1·70E-02 – 4·24E-04 |
| Dopamine degradation | B | 3/35 | 2·38E-03 | Cell cycle | SM | 14 | 1·84E-02 – 1·12E-04 |
| ERK5 signalling | SM | 4/63 | 6·17E-04 | Cell death and survival | L | 3 | 3·19E-02 – 1·70E-03 |
|  | P | 2/63 | 1·91E-02 |  | AV | 14 | 4·90E-02 – 3·90E-04 |
|  | AS | 4/63 | 1·03E-03 |  |  |  |  |
|  | AV | 3/63 | 1·23E-03 |  |  |  |  |
| Estrogen biosynthesis | L | 1/39 | 3·27E-02 | Cell morphology | B | 22 | 1·52E-02 – 1·11E-05 |
|  |  |  |  |  | AS | 30 | 3·48E-02 – 2·15E-03 |
|  | AV | 2/39 | 7·53E-03 |  | AV | 9 | 3·62E-02 – 2·44E-03 |
|  |  |  |  |  | P | 9 | 4·61E-02 – 3·08E-04 |
| Glycine betaine degradation | B | 2/10 | 2·50E-03 | Cell-to-cell signalling and interaction | L | 6 | 4·83E-02 – 8·53E-04 |
|  | AS | 2/10 | 2·15E-03 |  | T | 15 | 1·69E-02 – 7·15E-05 |
| HER-2 signaling in breast cancer | AS | 4/88 | 3·5E-03 | Cellular assembly and organisation | AS | 24 | 3·48E-02 – 2·17E-04 |
|  |  |  |  |  | AV |  |  |
|  |  |  |  |  | B | 38 | 1·52E-02 – 1·40E-06 |
| HIPPO signalling | SM | 4/86 | 1·97E-03 | Cellular compromise | T | 9 | 1·69E-02 – 4·24E-04 |
|  |  |  |  |  | AS | 12 | 3·48E-02 – 1·91E-03 |
| L-serine degradation | P | 1/3 | 1·01E-02 | Cellular development | B | 31 | 1·52E-02 – 1·40E-06 |
|  | AV | 1/3 | 1·08E-02 |  | L | 5 | 4·96E-02 – 8·53E-04 |
|  |  |  |  |  | P | 11 | 4·93E-02 – 9·88E-04 |
| Macropinocytosis signalling | AS | 4/81 | 2·61E-03 | Cellular growth and proliferation | B | 55 | 1·52E-02 – 1·40E-06 |
|  |  |  |  |  | L | 4 | 4·96E-02 – 8·53E-04 |
|  |  |  |  |  | AS | 10 | 3·48E-02 – 1·35E-04 |
| Melatonin degradation I | B | 5/62 | 1·10E-04 | Cellular movement | T | 8 | 1·69E-02 – 2·13E-04 |
|  | T | 3/62 | 1·57E-02 |  |  |  |  |
| Mitochondrial dysfunction | T | 5/171 | 1·54E-02 | Drug metabolism | B | 9 | 1·52E-02 – 1·28E-06 |
|  |  |  |  |  | SM | 4 | 1·84E-02 – 5·55E-04 |
|  |  |  |  |  | T | 3 | 1·69E-02 – 4·24E-04 |
| MSP-RON signalling | B | 4/59 | 1·07E-03 | Energy production | P | 3 | 2·33E-02 – 1·66E-04 |
| Notch signalling | L | 1/38 | 3·19E-02 | Lipid metabolism | AS |  |  |
|  |  |  |  |  | AV | 11 | 4·90E-02 – 7·11E-04 |
|  |  |  |  |  | P | 10 | 4·93E-02 – 1·66E-04 |
|  |  |  |  |  | SM | 18 | 1·57E-02 – 2·24E-04 |
| Oleate biosynthesis | SM | 2/13 | 2·81E-03 | Molecular transport | AV | 8 | 4·90E-02 – 1·64E-04 |
|  | T | 2/13 | 5·24E-03 |  |  |  |  |
|  |  |  |  |  | SM | 10 | 1·23E-02 – 5·55E-04 |
| Phosphatidylethanolamine biosynthesis III | P | 1/1 | 3·36E-03 | Post-translational modification | AS | 20 | 3·24E-02 – 3·23E-04 |
|  | T | 1/1 | 8·48E-03 |  |  |  |  |
|  | AV | 1/1 | 3·34E-03 |  |  |  |  |
| Role of cytokines in mediating communication between immune cells | L | 1/54 | 4·51E-02 | Small molecule biochemistry | P | 14 | 4·93E-02 – 1·66E-04 |
|  |  |  |  |  | SM | 24 | 1·84E-02 – 5·55E-04 |
|  |  |  |  |  | AV | 13 | 4·90E-02 – 3·90E-04 |
| Sertoli cell (junction) signalling | SM | 5/178 | 4·94E-03 |  |  |  |  |
| TNFR1 signalling | P | 2/49 | 1·18E-02 |  |  |  |  |
| Wnt/Ca+ pathway | L | 1/57 | 4·75E-02 |  |  |  |  |
| γ-linolenate biosynthesis II | SM | 2/17 | 4·82E-03 |  |  |  |  |
|  | T | 2/17 | 8·94E-03 |  |  |  |  |
| Dermatan sulfate biosynthesis | AV | 2/47 | 1·08E-02 |  |  |  |  |
| Virus entry via endocytic pathways | AS | 4/102 | 5·94E-03 |  |  |  |  |

AS, subcutaneous adipose; AV, visceral adipose; B, brain; L, liver; P, pancreas; SM, skeletal muscle; T, thyroid

**Supplementary Table 6.** Favourable adiposity eQTL SNPs from Yaghootkar et al ^2^ and their spatially regulated genes.

| SNP | Reported Gene | Interacting Gene | GTEx tissues | Effect size* |
| --- | --- | --- | --- | --- |
| rs2943645 | *IRS1* | *IRS1* | Subcutaneous adipose | -0·29 |
| rs731839 | *PEPD* | *PEPD* | Esophageal mucosa | 0·27 |
| rs974801 | *TET2* | *TET2* | Transformed fibroblasts | -0·16 |
| rs974801 | *TET2* | *PPA2* | Esophageal mucosa | -0·23 |
| rs974801 | *TET2* | *PPA2* | Subcutaneous adipose | -0·21 |
| rs4846565 | *LYPLAL1* | *SLC30A10* | Sun-exposed lower leg skin | -0·34 |
| rs780094 | *GCKR* | *GCKR* | Thyroid | 0·25 |
| rs780094 | *GCKR* | *FNDC4* | Thyroid | 0·30 |
| rs780094 | *GCKR* | *NRBP1* | Whole blood | -0·23 |
| rs780094 | *GCKR* | *NRBP1* | Subcutaneous adipose | -0·17 |
| rs780094 | *GCKR* | *NRBP1* | Sun exposed lower leg skin | -0·23 |
| rs780094 | *GCKR* | *NRBP1* | Testis | -0·29 |
| rs1530559 | *YSK4* | *AC016725.4* | Cerebellum | -0·52 |
| rs1530559 | *YSK4* | *TMEM163* | Whole blood | -0·29 |
| rs1530559 | *YSK4* | *TMEM163* | Testis | 0·37 |
| rs1530559 | *YSK4* | *TMEM163* | Esophageal mucosa | -0·35 |
| rs1530559 | *YSK4* | *DARS* | Thyroid | 0·23 |
| rs1530559 | *YSK4* | *CCNT2* | Aortal artery | 0·20 |

* Effect size of the eQTL SNP on the gene as provided by GTEx (version 4·1).

**References**

1. Rao, S. S. P. *et al.* A 3D Map of the Human Genome at Kilobase Resolution Reveals Principles of Chromatin Looping. *Cell* **159,** 1665–1680 (2014).

2. Yaghootkar, H. *et al.* Genetic Evidence for a Normal-Weight ‘Metabolically Obese’ Phenotype Linking Insulin Resistance, Hypertension, Coronary Artery Disease, and Type 2 Diabetes. *Diabetes* **63,** 4369–4377 (2014).
